# Pressure-driven membrane processes for the recovery and recycling of deep eutectic solvents: A seaweed biorefinery case study.

**DOI:** 10.1016/j.btre.2024.e00849

**Published:** 2024-06-25

**Authors:** Oscar M. Elizondo Sada, Isa S.A. Hiemstra, Nattawan Chorhirankul, Michel Eppink, Rene H. Wijffels, Anja E.M. Janssen, Antoinette Kazbar

**Affiliations:** a *Bioprocess Engineering*, Wageningen University & Research, PO Box 16 Wageningen 6700 AA, the Netherlands; b *Food Process Engineering*, Wageningen University & Research, PO Box 16 Wageningen 6700 AA, the Netherlands; c Nord University, *Faculty of Biosciences and Aquaculture*, N8049, Bodo, Norway

**Keywords:** Deep eutectic solvents, Membrane processes, Ultrafiltration, Diafiltration, Nanofiltration, Seaweed biorefinery

## Abstract

Deep eutectic solvents (DES) are green alternatives for conventional solvents. They have gained attention for their potential to extract valuable compounds from biomass, such as seaweed. In this framework, a case study was developed to assess the feasibility of pressure-driven membrane processes as an efficient tool for the recovery of deep eutectic solvents and targeted biomolecules. For this purpose, a mixture composed of the DES choline chloride – ethylene glycol (ChCl-EG) 1:2, water and alginate was made to mimic a DES extraction from seaweed. An integrated separation process design was proposed where ultrafiltration-diafiltration-nanofiltration (UF-DF-NF) was coupled. UF and DF were found to be effective for the separation of alginate with an 85 % yield. DES was likewise recovered by 93 %, proving the membrane filtrations’ technical feasibility. The NF performance to separate the DES from the water, for its recycling, laid by a 45 %-50 % retention and a final concentrated DES solution of 18 %(v/v).

## Highlights

• First time alginate was separated from DES using membrane processes.
• UF-DF was effective to separate alginate (85 %) and recover DES (93 %).
• Nanofiltration can be feasible for further DES purification from a water mixture.
• Proof of concept for a full membrane separation process for a seaweed biorefinery.

## Graphical abstract

**Figure:**
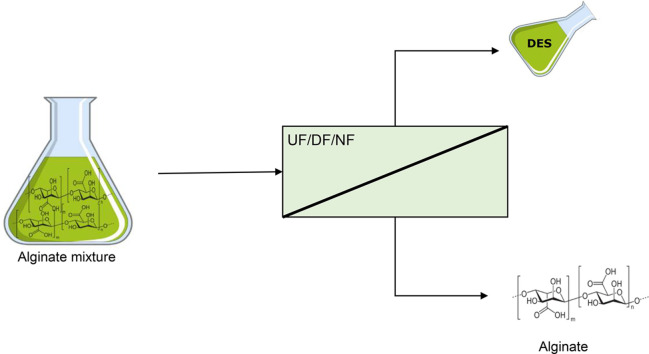

## 1 Introduction

The use of biomass to obtain bioproducts through biorefinery has become more feasible over the last years [1]. For sustainability reasons biorefineries must focus on the reduction of waste streams and hazardous chemicals employed [2]. Common extraction techniques still involve an unsustainable series of strong mineral acids and alkaline treatments [3]. To evade environmentally benign processes, new-age solvents such as deep eutectic solvents (DESs) have emerged as a promising alternative to conventional solvents, due to their sustainable nature and the possibility of intensifying processes by the prevention of mechanical cell disruption [4,5].

DESs are composed of two (or more) compounds (a hydrogen bond donor -HBD- and a hydrogen bond acceptor -HBA-) that form a eutectic mixture when added in a specific molar ratio [6]. When compared to conventional solvents or even earlier-considered “green” solvents as ionic liquids (ILs), DESs exhibit lower to no toxicity, have lower production costs, are renewable and biodegradable [7,8]. Furthermore, DESs have been praised for their excellent solvent capabilities, and their thermodynamic characteristics such as high thermal stability, low volatility, low vapor pressure, and tunable polarity [9,10]. As a result of their proven technical feasibility, DESs can be implemented for the development of sustainable and circular biorefineries [11,12]. Nevertheless, DESs are difficult to separate from their extracted products due to the low volatility and low vapor pressure and strong intramolecular interactions with the extracted products as well [13]. The challenge on back extraction limits the use of DESs in the industrial and commercial market, as recovery and recycling are important for the reduction of costs and carbon footprint [12].

Different separation processes have been explored using DES; however, the results have not been consistent, and several drawbacks limit their application and acceptance. Anti-solvent addition and crystallization have shown promising results, but incorporating additional unit operations, such as centrifugation and precipitation, during downstream processing not only complicates the process but also increases investment costs [14,15]. Supercritical CO_2_ and short path distillation, in addition to having high investment costs, are energy-intensive and have limited applications depending on the targeted molecule [16,17]. Limited application is also a challenge for liquid-liquid extraction and solid-liquid extraction, with the latter facing difficulties at scale-up due to the unavailability of microporous resins at a commercial scale [18].

Membrane processes has been applied in industry for the last decades due to its advantages over other separation processes [19]. These processes have a lower energy consumption, higher flexibility, lower cost-to-performance ratio, and favor more recycling when compared to other separation processes [20]. Membrane filtration can be hindered by fouling, as highlighted by Moslehyani et al., (2019) and Van der Bruggen et al. [21,22]. Fouling mechanisms, highly important during filtrations, fall into categories depending on whether they adhere to the membrane surface, deposit onto it, or block the pores within the membrane [23,24]. Cleaning membranes of foulants, achieved through physical or chemical methods, is therefore crucial [25,26]. While these topics are relevant, they are beyond the scope of this research, yet their mention remains pertinent. Nowadays, membrane processes have been listed as alternatives for the recovery and recycling of DESs [18]. Despite this potential, scarce research has been performed employing this type of separation process. In fact, only a coupled ultrafiltration and electrodialysis module reported in the work by Liang et al., (2019) where DES was recovered with a yield of 97 % [27]. However, electrical potential-driven processes such as electrodialysis have no ease of scale-up and are energy-intensive [28,29]. Nevertheless, this result highlights the potential application of membrane technology for the recovery and recycling of DESs, especially pressure-driven processes such as ultrafiltration and nanofiltration, which are used commercially in larger scale.

The aim of this study is to explore the use of a coupled membrane filtration system for the separation of a bioproduct from DES, while recovering the solvent. This is used as a case study and proof of concept, for the first time, under the seaweed biorefinery scope. Alginate was the target molecule, because it currently recovered from seaweed in an unsustainable process [30]. The DES selected was choline chloride – ethylene glycol 1:2 (ChCl-EG 1:2), as it has been reported to have significant extraction yield of alginate from brown seaweed [31]. Physiochemical parameters of this DES were assessed experimentally and via the software COSMO-RS. An ultrafiltration-diafiltration process was applied to separate alginate and recover the DES by washing it out. Different pressure levels, concentration factors and water content were evaluated for their impact on the membrane separations. A nanofiltration unit was further coupled to the process to concentrate the DES by permeating water through the membrane to explore the feasibility of stream recirculation.

## 2 Materials and methods

### 2.1 DES preparation

The DES ChCl-EG was prepared in a 1:2 molar ratio, by mixing choline chloride and ethylene glycol (Sigma Aldrich) at 80 °C in a water bath for 1 h until a translucid liquid was obtained. The molecular weight (MW) of this DES is 263.76 g mol^−1^. Due to the hygroscopic nature of choline chloride, the Metrohm KF Coulometer was used to verify the absence of water (<0.65 wt %) in the produced DES.

### 2.2 DES physicochemical characterization

The Anton Paar ViscoQC 300 Rotational Viscometer was used to measure the viscosity of the DES over a temperature range between 25 °C and 70 °C.

Viscosity can be described by the Arrhenius-type model, Eq. (1), which is the most used [32]. Here, η_0_ (mPas) is a constant, Ea (kJmol^−1^) the activation energy, *R* = 8.314 Jmol^−1^K^−1^ the ideal gas law constant, and T (K) the temperature.

(1) $$\eta =\;\eta_{0}e^{\frac{-Ea}{RT}}$$

**Fig. 1:**
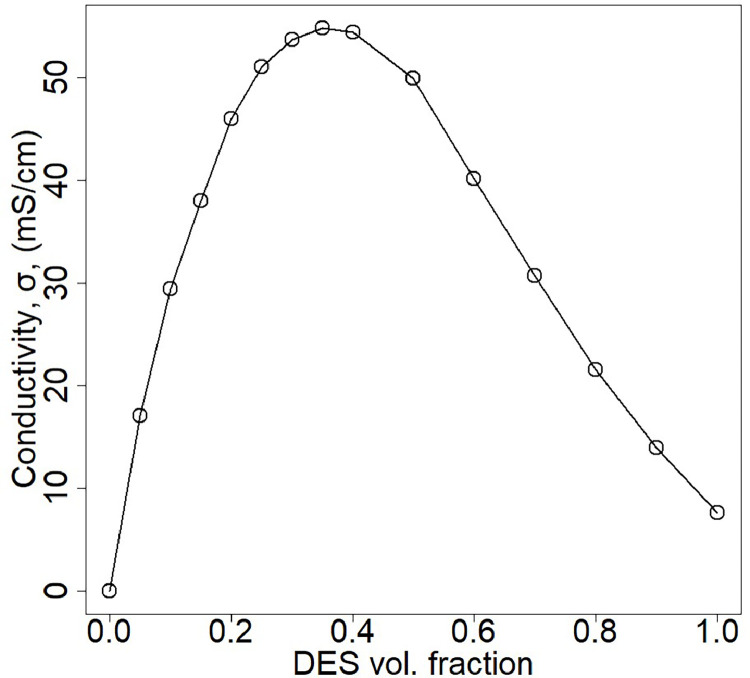
Conductivity as a function of the DES ChCl-EG 1:2 volumetric fraction.

The software COSMO-RS (Conductor-like Screening Model for Real Solvents) was used for the confirmation of the DES's affinity towards alginate, and for generating the activity coefficient plot. The Mettler Toledo Seven Compact Conductivity Meter was employed to measure the conductivity of the DES. Due to the known relationship between conductivity and DES fraction, it has been proposed as a fast and efficient method to quantify DES in streams [33,34]. The DES presence in the different streams was measured by correlating it with a DES ChCl-EG 1:2 conductivity standard calibration curve, shown in Fig. 1**.**

### 2.3 Sample preparation and alginate determination

Samples consisting of DES/H_2_O in 10 %, 25 %, 50 % (v/v%) were prepared to evaluate the effect of water during the membrane filtrations. Alginic acid sodium salt from brown algae (Sigma Aldrich) was added to each sample in a concentration of 1 gL^−1^ to mimic a mixture from an alginate extraction. Alginate determination was carried out following the 96-well plate uronic acid carbazole reaction proposed by Cesaretti et al. [35]. The reagents carbazole, sulfuric acid, sodium tetraborate, absolute ethanol and d-galacturonic acid were obtained from Sigma Aldrich.

### 2.4 Membranes

For the UF- DF experiments, a polyethersulfone (PES) ultrafiltration flat sheet membrane (LY series) from Synder Filtration™ with molecular weight cutoff (MWCO) of 100 kDa was used.

For the NF experiments, a spiral wound membrane (GE series) made of polyamide-TFC from SUEZ with a MWCO of 1000 Da and an effective membrane area of 0.37 m^2^ was employed.

### 2.5 Ultrafiltration and diafiltration set-up and experimental procedure

Dead-end ultrafiltration was performed in a pressurized and mechanically stirred set-up, utilizing a Millipore Stirred Cell (Amicon® Stirred Cell 400 mL) with an effective membrane area of 44.2 cm^2^. The stirrer velocity was kept constant throughout all experiments at 400 rpm (1.57 ms^−1^). The pressure levels of 1.4, 2.0, 2.8 bar were tested for each of the samples with a volume of 200 mL. The experiments ran for 120 min, which provided enough time to reach a steady-state condition. Analysis of variance (ANOVA) was performed to test the effects of the conditions. Before each experiment, the membranes were flushed with MilliQ water under pressure, and permeability tests were performed at the same pressure levels of all experiments. The UF/DFs were performed in the same set-up. The pressure was kept constant at 1.4 bar based on the preliminary UF experimental results. Three different volumetric concentration factors (VCF = 2, 3, 4) were investigated and MilliQ water was added as diafiltrate buffer to bring the total volume back to the original volume after each filtration. Diavolumes (N) were performed in each experiment until the conductivity in the permeate laid below 1 mScm^−1^, as that indicated that the DES concentration was insignificant and therefore washed-out. All tests were performed at room temperature. The permeate flux was calculated by first sampling over time and weighting on an Ohaus™ Explorer™ analytical balance and subsequently converted to volumes utilizing the density, as shown in Eq. (2):

(2) $$J=\;\frac{1}{A\rho }\frac{m}{\Delta t}$$

Where *J* is the permeate flux (Lm^−2^h^−1^), *A* is the effective membrane area (m^2^), *ρ* is the density (gL^−1^), *m* the weight of the permeate (g), and *Δt* the time the sample was collected (h).

The alginate rejection was defined by Eq. (3) [36]:

(3) $$R\;=\left[\frac{\ln\left(\frac{C_{R}}{C_{F}}\right)}{\ln\left(VCF\right)}\right]$$

Where R is the rejection of alginate expressed in percentage, C_F_ and C_R_ are the concentrations of alginate in the feed and retentate, respectively. VCF is the volumetric concentration factor (given by: $\frac{V_{F}}{V_{R}}$, where V_F_ and V_R_ are the volumes of the feed and retentate, respectively).

DES recovery was calculated following Eq. (4)**.**

(4) $$\%R_{DES\;}=\;\frac{V_{DES}^{F}}{\sum V_{DES}^{P}}*100$$

Where%R_DES_ is the recovery of the DES and$\;V_{DES}^{F}$ the volume of the DES in the initial feed solution and $\;V_{DES}^{P}\;$the volume of DES permeated from each stage of the UF/DF.

### 2.6 Nanofiltration set-up and experimental procedure

Nanofiltration of the resulting DES/H_2_O mixture post UF/DF was done in a continuous pilot and spiral wound set-up [37,38]. The feed solution, comprised of DES/H_2_O at a 6.4 % (v/v%) ratio, was circulated with an operational crossflow velocity of 0.1 ms^−1^. Pressure levels of 10, 15 and 20 bar were set. The temperature was held constant throughout the experiments at 25 ± 0.5 °C. Each condition ran for 45 min to reach steady state. After the time had elapsed, samples from the permeate and retentate were collected to analyze the DES fraction in each stream. Cleaning-in-place (CIP) was performed before and after the experiment following the cleaning procedure from Sealed Air, Diversey Care (The Netherlands).

## 3 Results and discussion

### 3.1 DES ChCl-EG 1:2 characterization

**Fig. 2:**
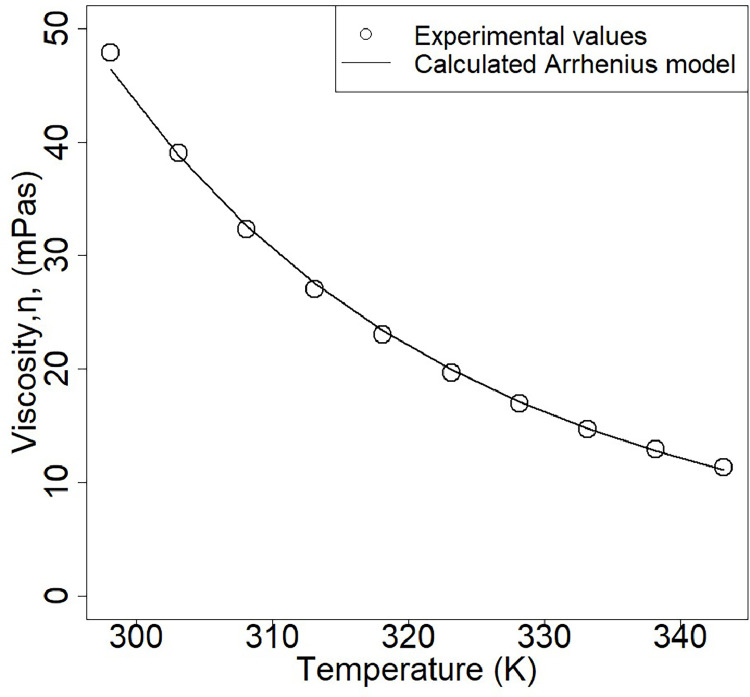
Experimental and calculated viscosity values for the DES ChCl-EG 1:2 over a range of temperatures.

The viscosity and activity coefficient of the DES ChCl-EG 1:2 were assessed for the membrane filtration case study. Viscosity can be one of the main roadblocks of DESs for practical purposes due to mass transfer limitations [39,40]. The viscosity of the DES ChCl-EG 1:2 showed no shear rate dependency on the viscosity, indicating Newtonian fluid behavior. Moreover, the relation between viscosity and temperature fitted validly within an Arrhenius model (Ea = −27.0 kJmol^−1^ and η_0_ = 8.5 × 10^−4^ mPas, see Fig. 2).

**Fig. 3:**
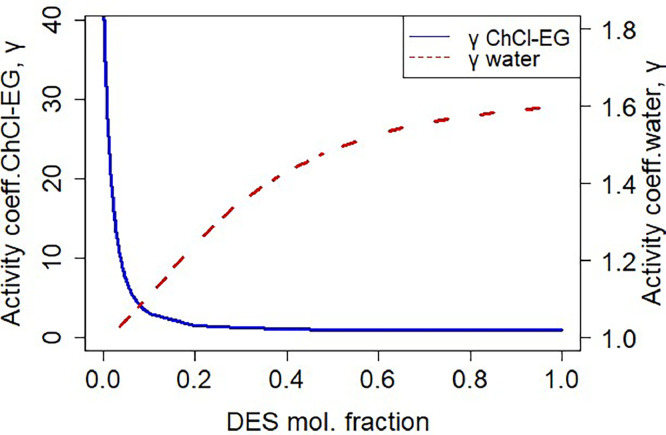
Activity coefficient of DES ChCl-EG 1:2 and water as a function of the DES mol fraction.

The viscosity of the DES at 25 °C (47.8 mPas) was found to be 50 times higher than that of water, thus posing possible mass transfer challenges during extraction. To improve mass transfer of alginate to the DES, either the temperature or the water content of the DES can be increased. The first option would require extra energy input for heating and the viscosity would still be 10-fold higher than water (Fig. 2**)**. On the contrary, the addition of 25 % water decreases the viscosity fivefold, and a fifteen times decrease is obtained when 50 % of water is added. This decrease in viscosity not only allows better mass transfer, but also it can avoid membrane fouling during the separation process [41]. Water addition does impact, however, the activity coefficient (γ) of the DES. The activity coefficient describes the thermodynamic intermolecular interactions, and is used for the evaluation of the extracting capacity of solvents [42,43]. For extraction operating conditions, γ values from 10 to 100 indicate weaker interactions, whereas values below 10 indicate strong interactions between solute and solvent [44]. The γ- value of ChCl-EG 1:2 in a mixture with water remains within practical purposes limits until the point where the mixture is composed of less than 0.01 mole fraction DES (Fig. 3). Therefore, for this study the DES_ChCl-EG 1:2_ /H_2_O ratios presented (Table 1) were selected to evaluate its effect on the membrane filtration performance.

**Table 1:** Viscosity of DES ChCl-EG 1:2 at different concentrations.

	DES concentration v/v% / (mol. fraction*, x*)			
	100 % (*1)*	50 % (*0.07)*	25 % (*0.04)*	10 % (*0.01)*
γ (-)	1.00	4.77	7.32	18.03
η (mPas)	47.82	3.20	1.60	1.12

### 3.2 Ultrafiltration and diafiltration

**Fig. 4:**
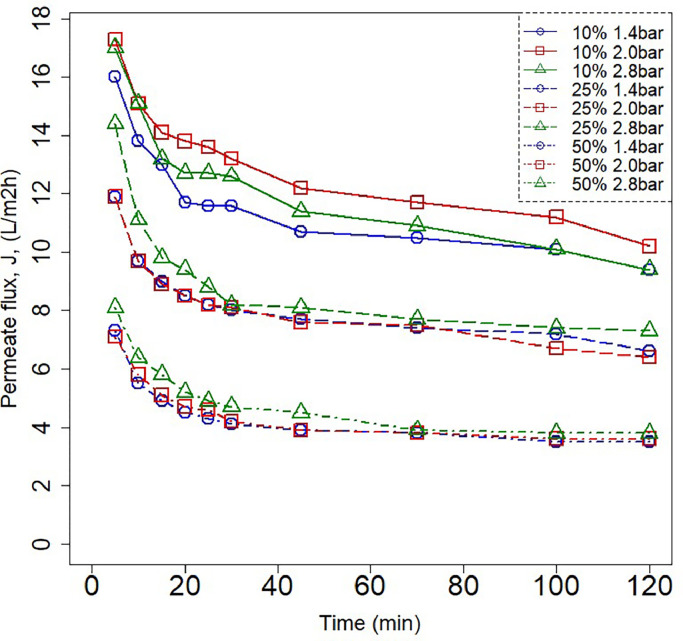
Permeate fluxes at 10 %, 25 % and 50 % DES/water ratios under three pressure levels. The PES membrane had a cut-off of 100 kDa.

Dead-end ultrafiltration was carried out to understand what the effects of the water content and the pressure are on the DES ChCl-EG 1:2 on the filtration performance when alginate is targeted to be retained. The permeability of the flat sheet membrane was found to be 64.1 ± 1.1 Lm^−2^ h^−1^ bar^−1^. The permeate flux behavior for each DES_ChCl-EG 1:2_ /H_2_O ratio with 1 gL^−1^ alginate is shown in Fig. 4. A significant decrease in the flux occurs as the DES concentration is increased. Nonetheless, the pressure levels do not affect the permeate flux (*p* > 0.05), thus an increase in pressure would not directly translate into a flux increase. The decrease of permeate flux as DES concentration increases can be explained by effects of an increase in the viscosity in the bulk of the feed solution and inside the membrane pores as well, as reported by Luo & Wan and Bowen & Yousef [45,46]. Which in accordance with Fick's Law and the Einstein Stokes relation, this will result in diffusion limitation through the membrane [47]. It can be hypothesized, as well, that a formation of a layer on the membrane surface, either by alginate or species of the DES, could have developed and hindered permeation through the membrane. The alginate rejection results, shown in Table 2**.**, indicate that alginate was successfully rejected. However, at a DES_ChCl-EG 1:2_ /H_2_O ratio of 50 % alginate rejection was lower compared to the other two concentration levels. This decrease in alginate rejection as the concentration of DES increases can be explained by the mass transfer mechanisms at the polarization layer. Freger [48] after observing this phenomenon, demonstrated that solute rejection decreases in the presence of salts [48]. This can be attributed to the Hofmeister effect, where the "salting-out" process causes partial dehydration of the solute [49]. This dehydration reduces the solute's hydrodynamic radius - Stokes radius -, leading to a decrease in solute retention [45]. Because a deep eutectic solvent is a liquid eutectic mixture of salts, in this presented work the addition of salt -DES- influences the retention of the solute -alginate- [50]. Additionally, Bargeman et al. [51] explained that the aforementioned “salting-out effect” causes the membrane pore size to increase because the hydration layer on pore walls becomes thinner [51]. They also proposed the presence of a pore size distribution, in which the Maxwell–Stefan model shows that the addition of salt with relatively low retention, in this present study the DES, reduces the flux through smaller pores and promotes more through the larger ones. Therefore, it can be strongly argued that the retention of alginate decreases as the DES concentration increases. These explanations align with the experimental ultrafiltration results. In their recent work, Aguirre Montesdeoca et al. [52] concluded that in fact, the observed rejection of all solutes decrease as the feed concentration increases, and fouling does not play a significant role in these cases [52]. This is due to the previously proposed pore size distribution effects and the non-idealities of the system at high concentrations at the polarization layer, which enhance flux and transport through larger pores.

**Table 2:** Alginate and DES rejections at different DES/water concentrations and pressures.

Alginate rejection	*1.4 bar*	*2.0 bar*	*2.8 bar*
*10 % DES/H_2_O*	0.95	0.95	0.96
*25 % DES/H_2_O*	0.92	0.93	0.94
*50 % DES/H_2_O*	0.82	0.81	0.84

Conductivity measurements were done to quantify the DES in the permeate stream and evaluate its recovery. For the 10 %, 25 % and 50 % samples, the DES recovery was 47.9 ± 2.7 %, 32.6 ± 0.9 % and 16.1 ± 1.4 %, respectively.

**Fig. 5:**
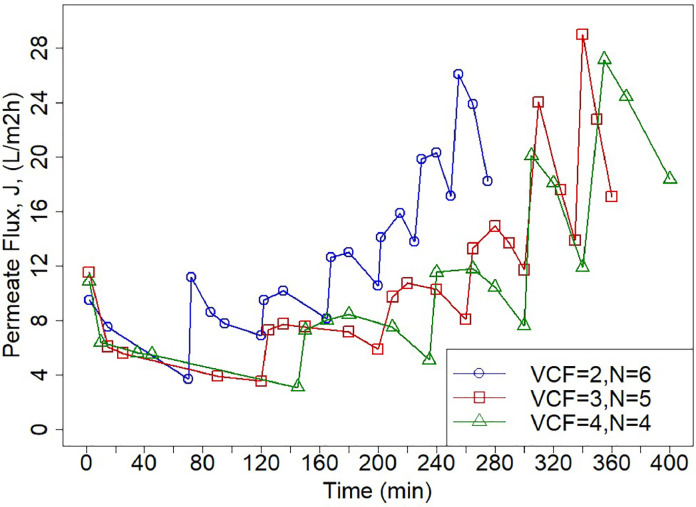
Ultrafiltration-diafiltration permeate fluxes over time.

**Fig. 6:**
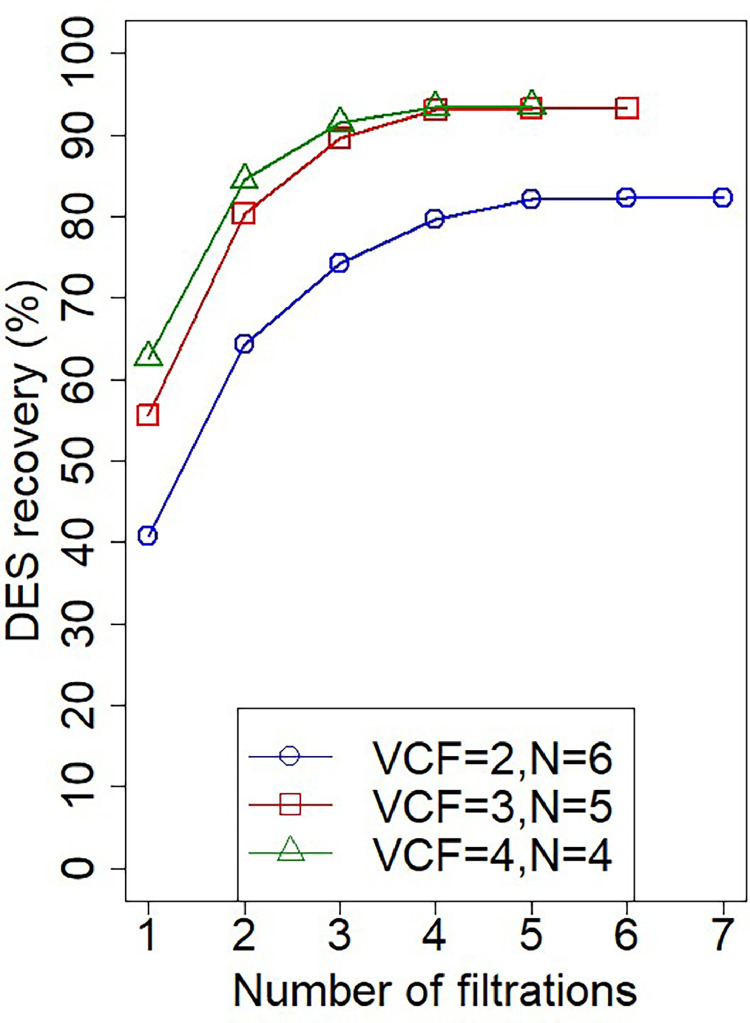
DES ChCl-EG 1:2 recovery as a function of filtration steps.

**Fig. 7:**
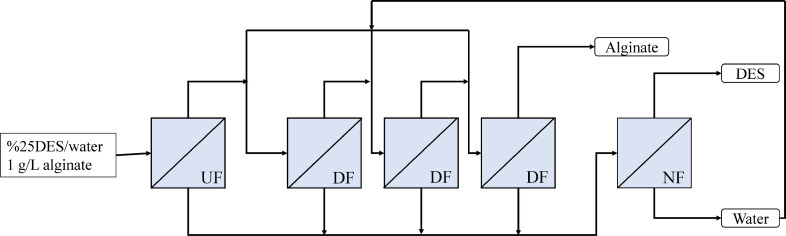
Proposed integrated separation process for alginate retention and DES recovery and recycling.

Based on the results, ultrafiltration-diafiltration (UF/DF) steps were carried out to fully recover the DES while retaining the alginate. The ultrafiltration works as a pre-concentration step, whereas the diafiltrations are targeted to wash out the DES. This type of configuration, involving DESs, has been reported previously by Gholami et al. [53]. For this coupled membrane process, the pressure was set at 1.4 bar, and a 25 % DES_ChCl-EG 1:2_ /H_2_O ratio was used. The latter was chosen because it theoretically resembles a pure DES extraction on fresh seaweed, which has a water content varying from 633 to 875 g per kg of wet weight [54]. Besides, Saravana et al. [31] reported that a 70 % water content in DES improves polysaccharide extraction yield from brown seaweed [31]. Furthermore, at a 25 % v/v DES_ChCl-EG 1:2_ /H_2_O, values for both viscosity and the activity coefficient are within operating conditions (Table 1). The volumetric concentration factor (VCF), defined in Section 2.6, and the number of diavolumes (N) were variables that were studied. Fig. 5 shows the behavior of the UF/DF permeate flux over time. Since the concentration of the DES decreases in every filtration step by the addition of water in each diavolume, the solution becomes less viscous, resulting in an increasing flux pattern of 1.9, 2.5, and 2.5 times for the VCF 2,3,4, respectively. The diavolume additions are graphically seen as the peaks in each of the curves. Conductivity measurements revealed that after 4 filtrations (UF - 3 DF), the maximum possible recovery of the DES was reached for the three of the conditions (82.2 %, 93.3 %, 93.5 %), as shown in Fig. 6. These results can also point towards fouling not being present. In the their most recent study, Gholami et al. [55] showed not only that the growth of the fouling layer reduces the recovery ratio for the second and third diafiltration steps, but also that higher solute and DES concentrations increase filtration resistance [55]. However, by the end of the diafiltration steps in this present study, the recovery of the DES was not hindered. This can be attributed to the concentration of both the DES and alginate being within a lower limit avoiding pore blockages and cake layer formation, as the mentioned study used a solute and DES concentration of 30 gL^−1^ and 70 %, respectively. Additionally, the rejection coefficient of the membrane for the DES was found to be 0.37±0.06. No difference between the experiments with a VCF of 3 and 4 was observed. However, for a VCF of 2, the recovery of the DES laid below 90 %. The diafiltration results regarding the variables of study are in agreement with previous studies on ultrafiltration based diafiltration [56,57]. With these experimental results, proving the efficacy of UF/DF for DES and alginate separation, a separation process design is proposed in Fig. 7. This overall process is a coupled cascade membrane filtration system with three final outlet streams. In this process, the feed solution enters a continuous ultrafiltration-diafiltration system with a working pressure of 1.4 bar. The ultrafiltration step is applied as a pre-concentration step to reduce the volume and to keep the alginate retained. The diafiltrations will dilute the previous retentate stream in order to permeate the DES ChCl-EG 1:2 and to retain alginate. The DF process with a VCF of 3 can be regarded as most suitable option out of the tested conditions. This is due to the high DES recovery yield (93.3 %) obtained here and the lower processing time required than a VCF of 4. After the UF/DF, the permeated total volume had a 6.4 % (v/v) DES_ChCl-EG 1:2_ /H_2_O concentration, thus being highly diluted. Therefore, a nanofiltration was coupled to the process design to further concentrate the DES and to possibly recover both streams.

### 3.3 Nanofiltration

Nanofiltration was performed in a pilot spiral wound single-stage configuration, graphically shown by [37]. The separation of the DES ChCl-EG 1:2 from water was studied. This allows for solvent recirculation, as well as reutilization of the water stream. The feed for the nanofiltration was a 6.4 % (v/v) DES_ChCl-EG 1:2_ /H_2_O solution permeated after the UF/DF. The permeability of the spiral wound membrane was 2.12 Lm^−2^h^−1^bar^−1^.

**Fig. 8:**
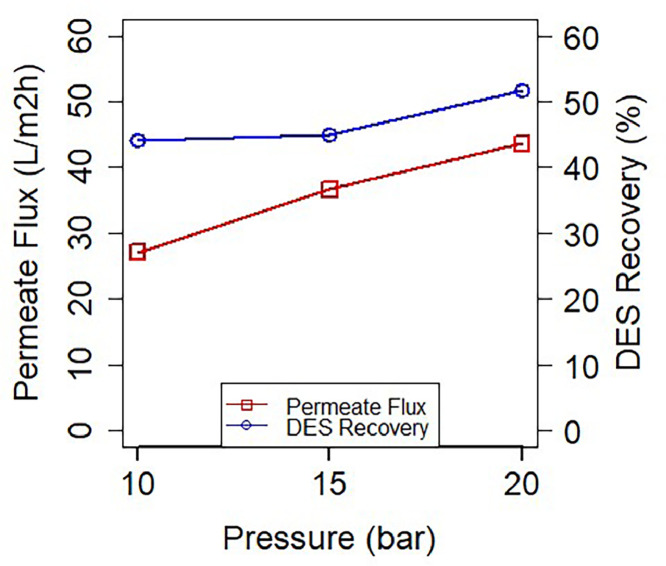
Permeate flux and DES recovery during the nanofiltration experiment. The process was configured in a pilot spiral wound set-up.

Fig. 8 shows the results from the NF experiments. The permeate flux increased linearly in each pressure level, meaning that the flux was not hindered in the studied pressure range. This finding is consistent with Wang et al. [58] where they showed that in their nanofiltration module the permeate flux increased linearly with the pressured applied [58].

After reaching steady state, the permeate conductivity was measured to quantify the DES that passed through the membrane. Retention of the DES was found to lay at 44.2 %, 45.0 % and 51.7 % for the pressure levels of 10, 15 and 20 bar, respectively. The retained DES ChCl-EG 1:2 had a final 18 % concentration (v/v%). This result concurs with the previously reported by Wang et al. [58] where it was managed to concentrate their solvent to 18.85 wt% from its initial content of 5 wt% utilizing a NF module [58]. Their study used ionic liquids (ILs) as solvent, and because DES are considered a new generation of ILs due to their similar physicochemical properties, results can be contrasted and discussed [59,60]. Abels et al. [33] also reported that at lower feed mass fractions, the applied pressure plays a main role in the separation of the solvent from the aqueous mixture [33]. In another study, Nakari et al. [61] found that the permeate fluxes can be increased by more than 50 % when the temperature was raised to 40 °C [61]. Thus, future research could focus on DES recovery with membranes while having temperature as a variable. Furthermore, Abels et al. [63] concluded that NF is effective to recover small molecular weight compounds, however for solvent recovery other membranes as electrodialysis (ED) must be employed, as illustrated by Liang et al. and Liang & Guo [27,62,63]. This remark is in accordance to Haerens et al. [34] where nanofiltration and a reverse osmosis (RO) module were used to separate the DES ChCl-EG (1:2), obtaining just a 20 % retention with the NF and up until 88 % with the RO [34]. The higher retention obtained in this current study compared to the previously mentioned, could be due to the dissociation of the DES into its components, and possibly the chlorine ion adsorbing to the membrane due to its negative charge.

## 4 Conclusion

The use of deep eutectic solvents in a commercial scale can be hindered by difficulties in the separation of extracted compounds, and the recovery of them. In this study, proof of concept was shown of ultrafiltration/diafiltration and nanofiltration to be an effective alternative for the separation and recovery of deep eutectic solvents and alginate. And for the first time a membrane filtration separation process design involving DESs was proposed for a seaweed biorefinery case study. The process consisted of coupled membrane filtrations (UF-DF-NF), with the aim of separating alginate from a DES/H_2_O mixture. Ultrafiltration and diafiltration effectively separated alginate with an 85 % yield from the DES, and the DES was recovered by 93 %. Nanofiltration underperformed to further purify the recovered DES, as the DES ChCl-EG 1:2 was retained to a final 18 % concentration (v/v%). Further research on the separation of DES from water is recommended and different pressures, temperatures, and MWCO are to be tested. Notwithstanding, this case study approach leads to conclude that there is technical feasibility for the use of pressure driven membrane processes for the separation of targeted biomolecules from DESs. Likewise, the use of membrane filtrations may be used for the recovery and recycling of deep eutectic solvents.

## Funding

This work was supported by the Dutch Research Council (NWO) [19479].

## CRediT authorship contribution statement

**Oscar M. Elizondo Sada:** Writing – original draft, Visualization, Validation, Project administration, Investigation, Formal analysis, Conceptualization. **Isa S.A. Hiemstra:** Visualization, Software, Investigation, Formal analysis, Data curation. **Nattawan Chorhirankul:** Writing – review & editing, Investigation. **Michel Eppink:** Writing – review & editing. **Rene H. Wijffels:** Writing – review & editing, Resources, Funding acquisition. **Anja E.M. Janssen:** Writing – review & editing, Supervision, Resources. **Antoinette Kazbar:** Writing – review & editing, Supervision, Project administration, Conceptualization.

## Declaration of competing interest

The authors declare that they have no known competing financial interests or personal relationships that could have appeared to influence the work reported in this paper.

## Data Availability

Data will be made available on request.
